# A human induced pluripotent stem cell line (TRNDi007-B) from an infantile onset Pompe patient carrying p.R854X mutation in the *GAA* gene

**DOI:** 10.1016/j.scr.2019.101435

**Published:** 2019-04-11

**Authors:** Yu-Shan Cheng, Rong Li, Amanda Baskfield, Jeanette Beers, Jizhong Zou, Chengyu Liu, Wei Zheng

**Affiliations:** a National Center for Advancing Translational Sciences, National Institutes of Health, Bethesda, MD, USA; b iPSC core, National Heart, Lung and Blood Institute, National Institutes of Health, Bethesda, MD, USA; c Transgenic Core, National Heart, Lung and Blood Institute, National Institutes of Health, Bethesda, MD, USA

## Abstract

Pompe disease is an autosomal inherent genetic disease caused by mutations in the *GAA* gene that encodes acid alpha-glucosidase. The disease affects patients in heart, skeletal muscles, liver, and central nervous system. A human induced pluripotent stem cell (iPSC) line was generated from the skin dermal fibroblasts of a Pompe patient with homozygosity for a C.2560C > T (p.R854X) mutation in exon 18 of the *GAA* gene. This human iPSC line provides a useful resource for disease modeling and drug discovery.

## Resource utility

This human induced pluripotent stem cell (hiPSC) line is a useful tool for studying disease pathophysiology and as a cell-based disease model for drug development to treat Pompe disease.

## Resource details

Pompe disease, also known as glycogen storage disease type II, is a rare autosomal recessive disorder caused by mutations in the *GAA* gene that results in a deficiency of lysosomal acid α-glucosidase (GAA). GAA facilitates the glycogen degradation by cleavage α-1,4 and α-1,6 linkages in glycogen. Deficiency in GAA results in accumulation of glycogen within lysosomes and in cytoplasm, eventually leading to tissue destruction. Infantile Pompe disease is the most severe subtype; disease symptoms occur in the first few months of life including muscle weakness, hypotonia, respiratory insufficiency and hypertrophic cardiomyopathy (Kishnani et al., 2006). On the other hand, late-onset Pompe disease may present at any age with slowly progressive proximal myopathy.

**Fig. 1.**
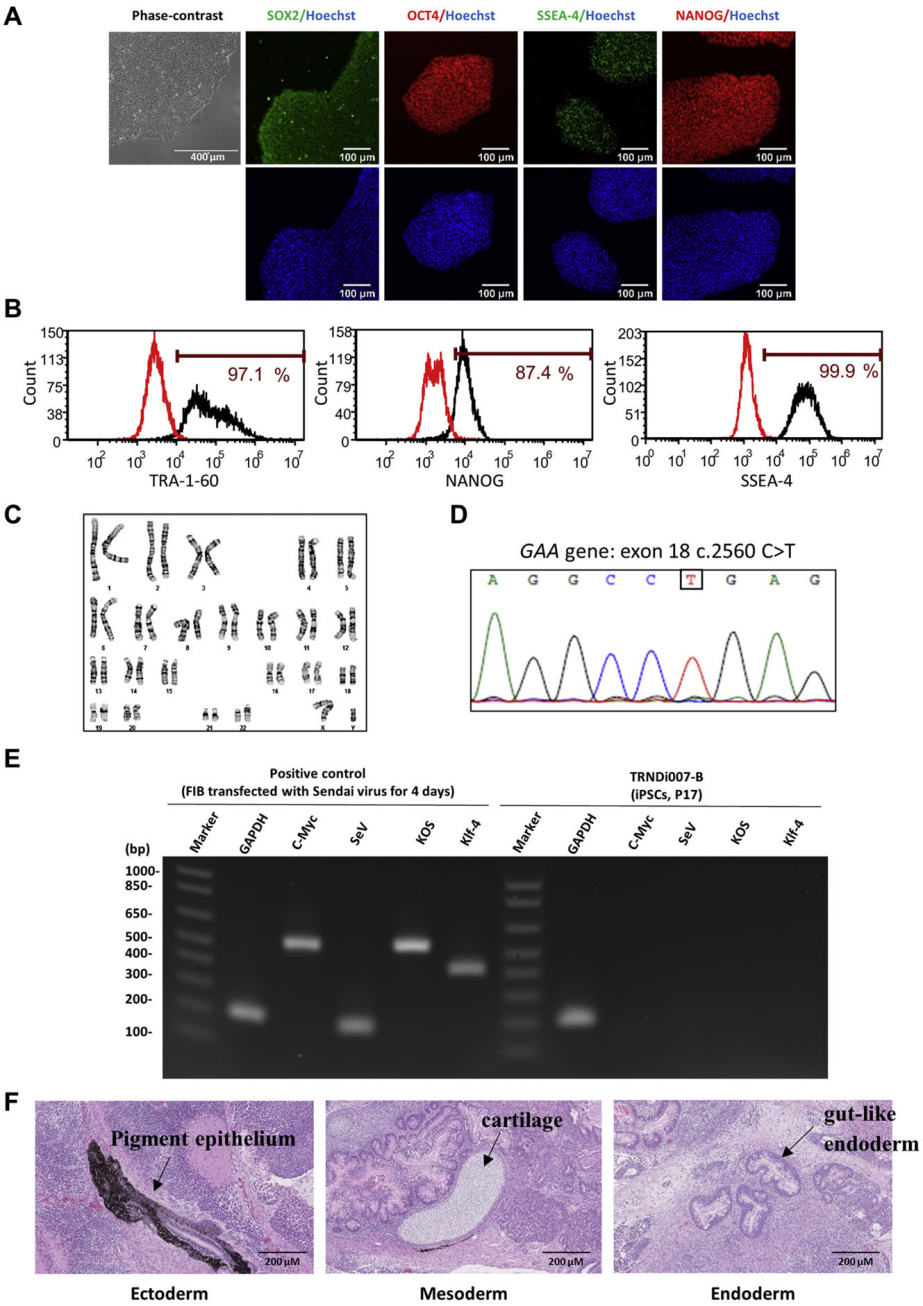
Characterization of TRNDi007-B iPSC line. **A)** Left: Phase contrast imaging of TRNDi007-B colonies. Right: Representative immunofluorescent images of iPSCs showing expression of stem cell markers: SOX2, OCT4, SSEA-4, and NANOG. Nucleus is stained with Hoechst dye (in blue). **B)** Flow cytometry analysis of pluripotency protein markers: TRA-1-60, NANOG and SSEA-4. **C)** Cytogenetic analysis showing a normal karyotype (46, XY). **D)** Detection of homozygous mutation of c.2560 C > T in exon 18 of the *GAA* gene. **E)** RT-PCR verification of the clearance of Sendai virus from the reprogrammed cells. Sendai virus vector transduced fibroblasts was used as positive control. **F)** Histological analysis of teratomas produced by TRNDi007-B iPSCs. Representative images showing the presence of ectodermal, endodermal and mesodermal derivatives.

In this study, we generated a human iPSC line (TRNDi007-B) from patient skin fibroblasts (GM00248) which was isolated from a five-month-old male patient carrying a homozygous mutation (c.2560C > T, p.R854X) in exon 18 of the *GAA* gene. R854X mutation is a well-documented African-American mutation and the homozygous mutation is responsible for severe infantile-onset Pompe disease (Becker et al., 1998). Patient fibroblasts were reprogramed into iPSC by transduction a set of non-integrating Sendai virus vectors encoding *OCT3/4, KLF4, S0X2 and C-MYC* genes (Beers et al., 2015). A single iPSC colony, termed TRNDi007-B, was isolated and characterized for future applications. Fig. 1A show that the iPSC line presents a standard iPSC morphology and expressed major pluripotent protein markers including SOX2, NANOG, OCT4, and SSEA-4 in the immunocytochemistry assay. The pluripotency markers including TRA-1-60, SSEA-4, and NANOG were also detected and quantified by the flow cytometry analysis (Fig. 1B). The mutation (c.2560C > T) in the GAA gene was confirmed by targeted Sanger sequencing where the PCR product harbors a single nucleotide variation (SNV) in both alleles (Fig. 1D). At passage 7, cells showed a normal human karyotype (46, XY) (Fig. 1C). The clearance of Sendai virus vectors and the exogenous reprogramming factor genes were verified by reverse transcription polymerase chain reaction (RT-PCR) at passage 17 (Fig. 1E). The iPSCs were free of mycoplasma (Supplementary Fig. S1) and the cell identity was proved by the short tandem repeat analysis (information available with the authors). Furthermore, the three-germ-layer differentiation capacity was demonstrated by the teratoma formation assay (Ectoderm, pigment epithelium; Mesoderm, cartilage; Endoderm, gut-like endoderm) *in vivo* (Fig. 1F).

## Materials and methods

### Cell culture

Human skin fibroblasts (GM00248) were obtained from the NIGMS Human Genetic Cell Repository at the Coriell Institute for medical research and maintained in DMEM containing 10% fetal bovine serum (HyClone), 100 units/mL penicillin, and 100 units/mL streptomycin. Human iPSCs were cultured in StemFlex medium (Thermo Fisher Scientific) on Geltrex-coated plates at 37 °C in humidified air with 5% CO_2_ and 5% O_2_. Dulbecco's Phosphate Buffered Saline (DPBS) containing 0.5mM ethylenediaminetetraacetic acid (EDTA) was employed during passaging (Table 1).

### Reprogramming of human skin fibroblasts

Patient fibroblasts were reprogrammed into iPSCs using a Sendai virus based kit (A16517, Thermo Fisher Scientific) as described previously (Beers et al., 2015).

### Mutation analysis

The genomic mutation analysis was carried out by Applied StemCell (Milpitas, CA). Genomic DNA was extracted using QuickExtract™ DNA Extraction Solution (Lucigen). PCR amplification was performed with MyTaq™ Red Mix (Bioline, Taunton, MA) and specific primers (Table 2). Amplifications were carried out on T00 Thermal Cycler (Bio-Rad #1861096) using the following condition: 2 min at 95 °C, 30 cycles of (15 s at 95 °C, 15 s at 60 °C, 15 s at 72 °C), and 72 °C for 5 min. The PCR products were Sanger sequenced.

### Immunocytochemistry

Cells were fixed in 4% paraformaldehyde for 20 min, rinsed with DPBS, and permeabilized with 0.5% Triton X-100/DPBS for 10 min at room temperature in a 96-well format. After blocked in cell staining buffer (BioLegend, CA) for 1 h at room temperature, cells were stained with primary antibodies/staining buffer (Table 2) for overnight at 4 °C. Cells were washed three times with DPBS and incubated with matched secondary antibodies (Table 2) for 1 h at room temperature. Cells were washed with DPBS again and stained with Hoechst 33342 for 20 min. After another wash, cells were imaged by an INCell Analyzer 2200 imaging system (GE Healthcare).

### Flow cytometry analysis

The iPSCs were dissociated using TrypLE Express enzyme (ThermoFisher Scientific), fixed with 4% paraformaldehyde for 10 mins at room temperature and then rinsed with DPBS. Before analysis, cells were permeabilized with 0.2% Tween-20 in DPBS for 10 min at room temperature and stained with fluorophore conjugated antibodies for 1 h at 4 °C on a shaker. Cells were then analyzed on a BD Accuri C6 Flow Cytometry system (BD Biosciences). Antibodies used are listed in Table 2.

### G-banded karyotyping

iPSCs were sent to WiCell Research Institute (Madison, WI) for G-banded karyotyping analysis. Experiments followed the standard cytogenetic protocol; results were concluded from 20 metaphase cells.

### Short tandem repeat (STR) analysis

STR profiling was conducted at WiCell Research Institute (Madison, WI) using PowerPlex 16 Kit (Promega).

### Mycoplasma test

Mycoplasma detection was performed using the MycoAlert mycoplasma detection kit (Lonza). Ratio B/A > 1.2 indicates mycoplasma positive. Ratio between 0.9-1.2 indicates ambiguous results. Ratio B/A < 0.9 indicates mycoplasma negative.

### Testing for Sendai reprogramming vector clearance

Total RNA was isolated by RNeasy Plus Mini Kit (Qiagen) and cDNA was synthesized from 1 μg of total RNA and random hexamers using Superscript™ III First-Strand Synthesis SuperMix (Thermo Fisher Scientific). PCRs were performed with Platinum II Hot-Start PCR Master Mix (Thermo Fisher Scientific) on Mastercycler pro S (Eppendorf) following a universal amplification setting: 94 °C for 2 min; 30 cycles of (94 °C for 15 s, 60 °C for 15 s) and 68 °C for 15 s. The primers are listed in Table 2. The positive control was derived from human fibroblasts (GM05659, Coriell Institute) infected with Sendai virus for four days.

### Teratoma formation assay

Teratoma was generated by a subcutaneous injection procedure. Approximately 1 × 10^7^ hiPSCs were constituted in 400 μL of 25mM HEPES (pH 7.4) solution. Prior to injection, cells were mixed with 200 μL of cold Matrigel (Corning, 354277). The mixture was injected subcutaneously into NSG mice (JAX No. 005557) at two sites (150 μL per injection site). After 6–8 weeks, visible tumors were removed and fixed in 10% Buffer Balanced Formalin. The fixed tumors were embedded in paraffin and stained with hematoxylin and eosin.

## Supplementary Material

1

## Figures and Tables

**Table 1:** Characterization and validation.

Classification	Test	Result	Data
Morphology	Photography	Normal	Fig. 1 Panel A
Phenotype	Immunocytochemistry	SOX2, OCT4, NANOG, SSEA-4	Fig. 1 Panel A
	Flow cytometry	TRA-1-60 (97.1%); NANOG (87.4%); SSEA-4 (99.9%)	Fig. 1 Panel B
Genotype	Karyotype (G-banding) and resolution	46XY Resolution: 350–400	Fig. 1 Panel C
Identity	Microsatellite PCR (mPCR) OR	Not performed	N/A
	STR analysis	16 sites tested, all sites matched	Available with the authors
Mutation analysis (IF APPLICABLE)	Sequencing	Homozygous mutation of *GAA* C.2560C > T in exon 18	Fig. 1 Panel D
	Southern Blot OR WGS	N/A	N/A
Microbiology and virology	Mycoplasma	Mycoplasma testing by luminescence. Negative	Supplementary Fig. S1
Differentiation potential	Teratoma formation	Teratoma with three germlayers formation. Ectoderm (pigment epithelium); Mesoderm (cartilage); Endoderm (gut-like endoderm)	Fig. 1 Panel F
Donor screening (OPTIONAL)	HIV 1+2 Hepatitis B, Hepatitis C	N/A	N/A
Genotype additional info (OPTIONAL)	Blood group genotyping	N/A	N/A
	HLA tissue typing	N/A	N/A

**Table 2:** Reagents details.

Antibodies used for immunocytochemistry/flow-citometry			
	Antibody	Dilution	Company Cat # and RRID
Pluripotency Markers	Mouse anti-SOX2	1:50	R&D Systems, Cat# MAB2018, RRID:AB_358009
Pluripotency Markers	Mouse anti-SSEA-4	1:1000	Cell Signaling Technology, Cat# 4755, RRID:AB_1264259
Pluripotency Markers	Rabbit anti-NANOG	1:400	Cell Signaling Technology Cat# 4903, RRID:AB_10559205
Pluripotency Markers	Rabbit anti-OCT4A	1:400	Thermo Fisher Cat# A13998, RRID:AB_2534182
Secondary antibodies	Donkey anti-Mouse IgG (Alexa Fluor 488)	1:400	Thermo Fisher, Cat# A21202, RRID: AB_141607
Secondary antibodies	Donkey anti-Rabbit IgG	1:400	Thermo Fisher, Cat# A21207, RRID: AB_141637
Flow cytometry antibodies	Anti-Tra-1-60-DyLight 488	1:50	Thermo Fischer, Cat# MA1–023-D488X, RRID: AB_2536700
Flow cytometry antibodies	Anti-Nanog-Alexa Fluor 488	1:50	Millipore, Cat# FCABS352A4, RRID: AB_10807973
Flow cytometry antibodies	Anti-SSEA-4-Alexa Fluor 488	1:50	Thermo Fischer, Cat# 53–8843-41, RRID: AB_10597752
Flow cytometry antibodies	Mouse-IgM-DyLight 488	1:50	Thermo Fischer, Cat# MA1–194-D488, RRID: AB_2536969
Flow cytometry antibodies	Rabbit IgG-Alexa Fluor 488	1:50	Cell Signaling, Cat# 4340S, RRID: AB_10694568
Flow cytometry antibodies	Mouse IgG3-FITC	1:50	Thermo Fischer, Cat# 11–4742-42, RRID: AB_2043894
Primers			
	Target		Forward/Reverse primer (5′-3′)
House-Keeping Genes (RT-qPCR)	*GAPDH/197bp*		Fw: GGA GCG AGA TCC CTC CAA AAT Rv: GGC TGT TGT CAT ACT TCT CAT GG
Sev specific primers (RT-qPCR)	*SeV/181bp*		Fw: GGA TCA CTA GGT GAT ATC GAG C Rv: ACC AGA CAA GAG TTT AAG AGA TAT GTA TC
Sev specific primers (RT-qPCR)	*KOS/528bp*		Fw: ATG CAC CGC TAC GAC GTG AGC GC Rv: ACC TTG ACA ATC CTG ATG TGG
Sev specific primers (RT-qPCR)	*Klf4/410bp*		Fw: TTC CTG CAT GCC AGA GGA GCC C Rv: AAT GTA TCG AAG GTG CTC AA
Sev specific primers (RT-qPCR)	*C-Myc/523bp*		Fw: TAA CTG ACT AGC AGG CTT GTC G Rv: TCC ACA TAC AGT CCT GGA TGA TGA TG
Targeted mutation analysis (PCR)	*GAA/834bp*		Fw: CCC GCA GTG TAG GTT ATC AAG G Rv: CTC CCT CAC TGG TCA CAC GTA C

**Table:** Resource table.

Unique stem cell line identifier	TRNDi007-B
Alternative name(s) of stem cell line	HT521B
Institution	National Institutes of Health National Center for Advancing Translational Sciences Bethesda, Maryland, USA
Contact information of distributor	Dr. Wei Zheng Wei.Zheng@nih.gov
Type of cell line	iPSC
Origin	Human
Additional origin info	Age: five-month Sex: Male Ethnicity: African American
Cell source	Skin fibroblasts
Clonality	Clonal
Method of reprogramming	Integration-free Sendai viral vectors
Genetic modification	NO
Type of modification	N/A
Associated disease	Pompe disease
Gene/locus	GAA, chromosomal location:17q25.2-q25.3, genotype: exon 18, C.2560C > T (p.R854X)
Method of modification	N/A
Name of transgene or resistance	N/A
Inducible/constitutive system	N/A
Date archived/stock date	2017
Cell line repository/bank	N/A
Ethical approval	NIGMS Informed Consent Form was obtained from patient at time of sample submission. Confidentiality Certificate: CC-GM-15-004

## References

[R1] Becker JA, Vlach J, Raben N, Nagaraju K, Adams EM, Hermans MM, Reuser AJ, Brooks SS, Tifft CJ, Hirschhorn R, Huie ML, Nicolino M, Plotz PH, 1998. The African origin of the common mutation in African American patients with glycogen-storage disease type II. Am. J. Hum. Genet. 62, 991–994. 9529346 10.1086/301788 PMC1377028

[R2] Beers J, Linask KL, Chen JA, Siniscalchi LI, Lin Y, Zheng W, Rao M, Chen G, 2015. A cost-effective and efficient reprogramming platform for large-scale production of integration-free human induced pluripotent stem cells in chemically defined culture. Sci. Rep. 5, 11319. 26066579 10.1038/srep11319 PMC4464084

[R3] Kishnani PS, Steiner RD, Bali D, Berger K, Byrne BJ, Case L, Crowley JF, Downs S, Howell RR, Kravitz RM, Mackey J, Marsden D, Martins AM, Millington DS, Nicolino M, O'Grady G, Patterson MC, Rapoport DM, Slonim A, Spencer CT, Tifft CJ, Watson MS, 2006. Pompe disease diagnosis and management guideline. Genet. Med. 8, 267–288. 16702877 10.1097/01.gim.0000218152.87434.f3 PMC3110959

