# Supplementary figures and images for: A human induced pluripotent stem cell line (TRNDi007-B) from an infantile onset Pompe patient carrying p.R854X mutation in the GAA gene
Source: Stem Cell Res. Author manuscript; Available in PMC 2019 Jul 25. (PMC6658133; doi:10.1016/j.scr.2019.101435)

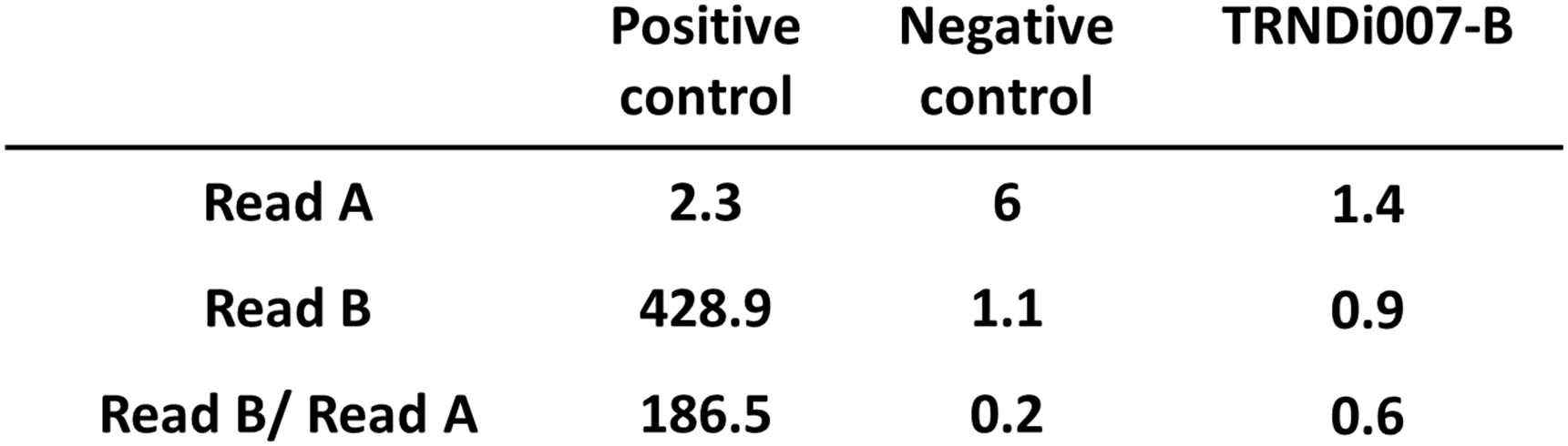

Supplement: 1 [file NIHMS1530904-supplement-1.jpg]
